# Left circumflex coronary artery injury following mitral valve replacement with late presentation: A case report and literature review

**DOI:** 10.34172/jcvtr.2022.30551

**Published:** 2022-12-31

**Authors:** Zahra Nassereddine, Hassan Kazem, Malek Moussa, Mohamad Saab

**Affiliations:** ^1^Beirut Cardiac Institute, Cardiology Division, Beirut, Lebanon; ^2^Beirut Cardiac Institute, Cardiovascular and Thoracic Surgery Division, Beirut, Lebanon

**Keywords:** Mitral Valve Surgery, Coronary Ischemia, Percutaneous Intervention

## Abstract

Mitral valve replacement complications are more and more recognized due to the novel surgical techniques and the tendency to report such complications by young cardiologists and surgeons. Circumflex coronary artery injury is a rare complication that occurs during mitral valve replacement or repair by multiple mechanisms. We present the case of a 57-year-old female who underwent mitral valve replacement and ended up with heart failure after circumflex artery occlusion and failure of percutaneous coronary intervention.

## Introduction

 Left circumflex coronary artery (LCX) injury during mitral valve repair and replacement is a rare but well-known complication due to the proximity of the artery to the posterior mitral annulus.^1^ Limited case reports were published suggesting multiple risk factors and multiple mechanisms. The resultant myocardial ischemia is evident usually intraoperatively or in early post operative period in most cases.^2^ The treatment includes both surgery or percutaneous coronary intervention (PCI) and showed different results. Here we present the case of a lady who had symptoms of heart failure few weeks after mitral valve replacement in our center and found to have occluded proximal LCX. We discuss the available knowledge concerning this rare surgical complication and how to avoid and to treat in light of available data in literature.

## Case Presentation

**Figure 1:**
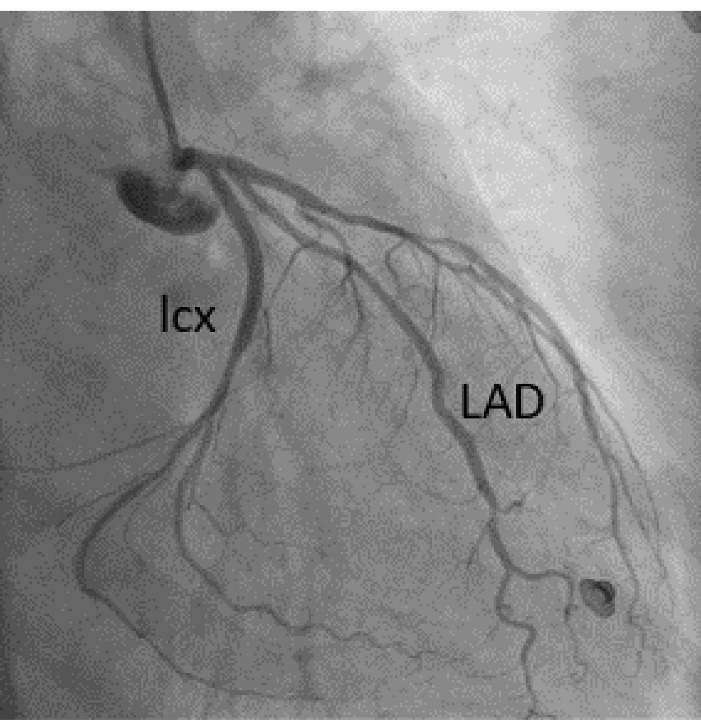
Coronary angiography RAO caudal view; a moderate stenosis on mid LAD is seen, RAO; right anterior oblique, LAD; left anterior descending artery.

**Figure 2:**
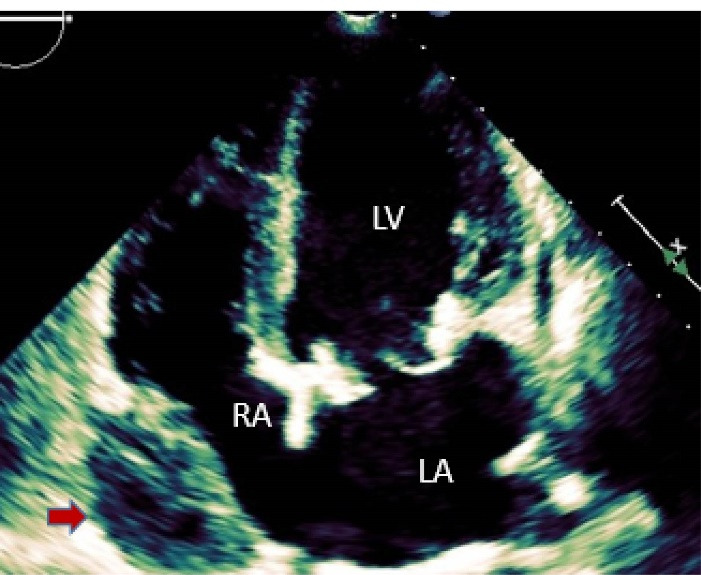
Transthoracic echocardiography, apical 4 chambers view: A hematoma around the right atrium is seen (red arrow).

**Figure 3:**
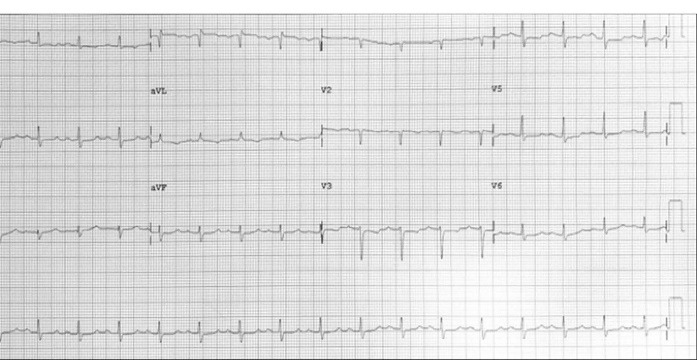
EKG four weeks later showed non specific ST-T changes.

**Figure 4:**
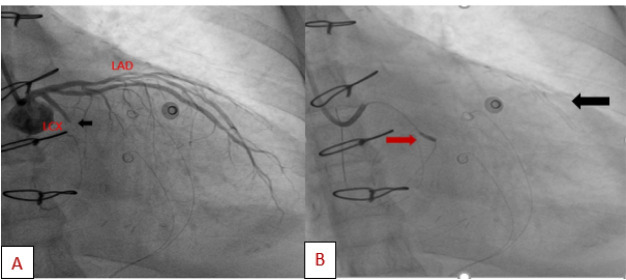
(A) Coronary angiography shows proximal LCX stenosis (black arrow). (B) Trial of PCI of the circumflex coronary artery, the balloon (red arrow) had crossed easily but failed to dilate the lesion. Successful angioplasty of LIMA-LAD stenosis, stent is shown (black arrow), PCI; percutaneous coronary intervention.

 A 57-year-old lady with history of rheumatic mitral valve disease underwent mitral valve replacement by Saint Jude Medical Epic bioprosthesis in our high-volume mitral surgery center. Preoperative coronary angiography was non-significant except for a moderate stenosis of mid left anterior descending artery (LAD) (Figure 1). The patient underwent coronary artery bypass grafting (CABG) using left internal mammary artery (LIMA) to LAD concomitantly with mitral valve surgery. The surgery was smooth with no postoperative complications. On serial postoperative electrocardiogram (EKG), there was no ST segment elevation or new significant ischemic changes compared to preoperative EKG. Routine postoperative echocardiogram showed good function of the mitral prosthesis and good global function of the left ventricle. Four weeks later, the patient started to have dyspnea, orthopnea and other symptoms of heart failure. EKG showed non-significant ST-T changes (Figure 2). Echocardiography showed severely impaired left ventricle (LV) systolic function. Moreover, there was a localized hematoma adjacent to the right atrium without hemodynamic impact (Figure 3). In light of these findings along with elevated troponin level, we decided to repeat coronary angiography. The later showed complete occlusion of the proximal LCX (Figure 4A) and stenosis of the LIMA -LAD anastomosis. A PCI strategy was successful for LIMA-LAD stenosis (Figure 4B) but difficult for LCX lesion; after crossing the lesion using work-horse wire, and use of different size non-compliant and compliant balloons, there was continuous recoil after balloon deflation (Figure 4B). The trial of angioplasty and stenting was failed with no recuperation of distal flow. Therefore, we suspected surgical complication with mechanical compression of the circumflex coronary artery most probably by encircling suture loop, in addition to stenosis of the LIMA-LAD. Furthermore, the retro atrial hematoma might be caused by epicardial coronary artery injury. The procedure was then stopped and the patient was kept on medical treatment. On follow up visits, there was complete resolution of the atrial hematoma, and a significant improvement of LV function on standard heart failure treatment.

## Discussion

 Both mitral valve repair and replacement can be complicated rarely by LCX injury.

 The mechanisms include mechanical: encircling suture, direct laceration and resultant thrombotic occlusion, external compression by subintimal hematoma or kinking of the artery, or non-mechanical complications: coronary vasospasm or local thrombosis or artery embolism (air, bone marrow, suture materiel...).^2^ In our case, we suggest that the mechanism of LCX injury was an encircling suture loop or external epicardial hematoma, this complication was initially silent and along with LIMA-LAD stenosis leads to left ventricle dysfunction.

 The risk of LCX injury during mitral valve surgery is increased in patient with left dominance and codominance due to the proximity of the vessel to the mitral annulus.^3,4^ The mean distance to mitral annulus was determined in a direct examination of 15 hearts done by Virmani et al shorter distance was noted in left dominance compared to other variants; 4.1 mm versus 5,5 mm in codominance and 8.4 mm in right dominance.^3^ Same findings were also seen by Caruso et al in a in vivo study of anatomical zones where the distance between mitral annulus and LCX were measured on coronary computed tomography angiography (CCTA) of 95 patients.^5^ Carusu et al suggest using preoperative CCTA in identifying patients at high risk of LCX injury post mitral valve surgery. The most vulnerable part of the artery was the proximal third due to close relation to the annulus.^3^ A comprehensive review by Hiltrop et al^6^ suggests some risk factors associated with this rare complication in addition to left dominant coronary system; anomalous circumflex artery arising from the right coronary cusp,^2^ reoperations, and other procedure related factors such as extensive decalcification, aggressive leaflet resections, the use of non-undersized annuloplasty rings and concomitant procedures including closure of left atrial appendage and atrial fibrillation ablation.^6^ Furthermore, the technique of minimally invasive mitral valve surgery may be associated with increased risk of circumflex artery injury compared to conventional surgery.^6^ In fact, we noted that most cases reported in literature had this complication after minimally invasive mitral valve surgery as compared to fewer cases after classical surgery.^1,2,6^ We suggest avoiding minimally invasive surgery in high-risk patients.

 Intraoperative EKG monitoring for any ischemic changes or arrythmias and routine use of intraoperative transesophageal echocardiogram (TEE) to detect new regional wall motion abnormalities are important during mitral valve replacement as well as during mitral valve repair. Moreover, electrocardiograms should be obtained systematically post mitral valve surgery the same day of surgery. Coronary ischemia is suspected on EKG with ST elevation or tachyarrhythmias and less frequently on intraoperative transesophageal echocardiography or postoperative echocardiography with appearance of new regional wall motion abnormalities, usually after weaning of cardiopulmonary bypass in the operating room or after transfer to intensive care units. Actually, most reported cases were diagnosed in the operating room, less frequently within days,^6^ and rarely, the diagnosis can be delayed for weeks such as in our case according to the underlying mechanism. Probably, the coronary injury started at the time of surgery and progressed over the following days. Urgent coronary angiography is then indicated to detect LCX injury earlier post mitral valve surgery.

 Primary PCI was tried in most cases. Some cases described in literature showed good results with successful angioplasty,^7-9^ while other cases were complicated by coronary perforation or premature stent restenosis or transferred to operating room for surgical intervention after failed percutaneous intervention.^10,11^ A conservative approach was adopted in minority of cases where the artery was of small caliber in absence of hemodynamic or electrical instability. Surgical revision was done when coronary ischemia was suspected before chest closure or in some instable patients in immediate postoperative period.

## Conclusion

 We presented a case of mitral valve surgery complication diagnosed 4 weeks post op; Injury to the LCX artery is a rare complication of mitral valve replacement or repair that can be silent initially. The cardiovascular team can determine high risk patients to avoid minimally invasive techniques and to be careful for signs of coronary ischemia in these patients during the immediate postoperative period as well as during the following weeks.

## Acknowledgments

 To Mrs Lina Hneineh. Thanks for helping in patient follow up and visits.

## Author Contributions

**Conceptualization:** Zahra Nasserddine.

**Methodology:** all authors.

**Validation:** All authors contribute to validate the work.

**Formal Analysis:** Zahra Nassereddine.

**Investigation:** All authors.

**Resources:** All authors.

**Data Curation:** All authors.

**Writing—Original Draft Preparation:** Zahra Nassereddine.

**Writing—Review and Editing:** Zahra Nassereddine.

**Visualization:** Zahra Nasseredine.

**Supervision:** Mohamad Saab.

**Project Administration:** Mohamad Saab.

## Funding

 None.

## Ethical Approval

 An informed consent was obtained from the patient.

## Competing Interest

 The authors declare that there is no conflict of interest.
